# Major roles of kupffer cells and macrophages in NAFLD development

**DOI:** 10.3389/fendo.2023.1150118

**Published:** 2023-05-19

**Authors:** Soo-Jeung Park, Josefina Garcia Diaz, Eugene Um, Young S. Hahn

**Affiliations:** ^1^ Beirne B. Carter Center for Immunology Research, University of Virginia, Charlottesville, VA, United States; ^2^ Department of Microbiology, Immunology and Cancer Biology, University of Virginia, Charlottesville, VA, United States

**Keywords:** non-alcoholic fatty liver disease (NAFLD), liver, immune system, kupffer cells, macrophages, therapeutics

## Abstract

Non-alcoholic fatty liver disease (NAFLD) is an important public health problem with growing numbers of NAFLD patients worldwide. Pathological conditions are different in each stage of NAFLD due to various factors. Preclinical and clinical studies provide evidence for a crucial role of immune cells in NAFLD progression. Liver-resident macrophages, kupffer cells (KCs), and monocytes-derived macrophages are the key cell types involved in the progression of NAFLD, non-alcoholic steatohepatitis (NASH), and hepatocellular carcinoma (HCC). Their unique polarization contributes to the progression of NAFLD. KCs are phagocytes with self-renewal abilities and play a role in regulating and maintaining homeostasis. Upon liver damage, KCs are activated and colonized at the site of the damaged tissue. The secretion of inflammatory cytokines and chemokines by KCs play a pivotal role in initiating NAFLD pathogenesis. This review briefly describes the role of immune cells in the immune system in NAFLD, and focuses on the pathological role and molecular pathways of KCs and recruited macrophages. In addition, the relationship between macrophages and insulin resistance is described. Finally, the latest therapeutics that target KCs and macrophages are summarized for the prevention and treatment of NAFLD.

## 1 Introduction

**Figure 1:**
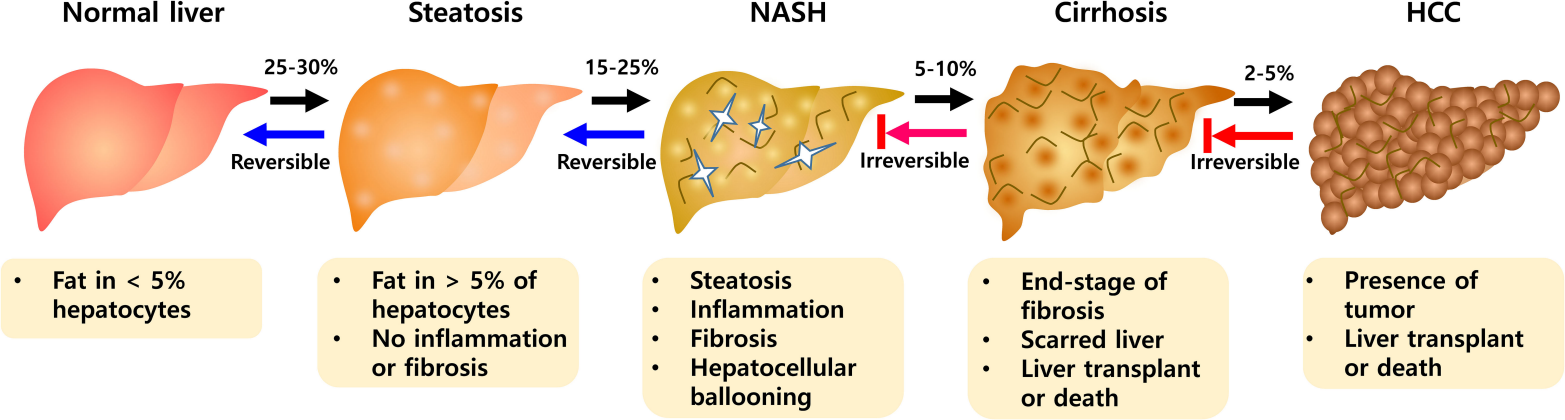
The progression of NAFLD pathogenesis. During NAFLD, hepatic abnormalities develop from a steatosis to NASH evolving toward fibrosis. NASH is progressive state that can advance to Cirrhosis and HCC. NASH, non-alcoholic steatohepatitis; HCC, hepatocellular carcinoma.

A recent systematic review and meta-analysis reported the worldwide prevalence of non-alcoholic fatty liver disease (NAFLD) at approximately 25.2% to 29.8% (1). Predictions indicated that more than 300 million people in China, more than 100 million people in the United States, and 15 to 20 million people in major European countries will suffer from NAFLD by 2030 (2). NAFLD is associated with obesity, type 2 diabetes, dyslipidemia, and metabolic syndrome, and is an important public health disease (3, 4). It is also known as a major problem for liver transplantation (5) and includes various stages such as steatosis, non-alcoholic steatohepatitis (NASH), hepatic fibrosis, and hepatocellular carcinoma (HCC) (6). The presence of steatosis along with symptoms such as hepatocyte damage and lobular inflammation defines NASH with progressive fibrosis (**Figure 1**). NASH is a key step from the progression of NAFLD because of the potential for further development to end-stage liver diseases such as cirrhosis and hepatocellular carcinoma (HCC) (7). NASH is a complex disease with multiple causes and the exact etiology is not fully understood. Steatosis is an early onset of NAFLD, but does not necessarily lead to NASH (6). As part of the pathogenesis of NASH, there are major mechanisms including insulin resistance, hepatic lipid accumulation, endoplasmic reticulum stress, development of dyslipidemia and inflammation (8–10).

The liver is an important organ for metabolic regulation and has multiple immune cells. In particular, the innate immune system, in addition to its role in detecting foreign substances, promotes metabolic disorders during NAFLD progression (11). The liver contains a network of innate immune cells, such as neutrophils, monocytes, kupffer cells (KC), dendritic cells (DC), natural killer cells (NK), innate lymphoid cells (ILC), invariant NKT cells (iNKT), and mucosal-associated invariant T cells (MaITs) (12, 13). They act as the first line of defense against environmental changes and invading pathogens. These immune cells play a major role in promoting inflammation by releasing chemokines, cytokines, eicosanoids, reactive oxygen species, and nitric oxide (14, 15). In addition, there are molecules involved in effector function of innate immune cells in NAFLD such as pattern-recognition receptors (PRRs; e.g., toll-like receptors, NOD-like receptors, oligoadenylate synthase-like receptors, and advanced glycation end-products and receptor of AGE) (16). Receptor binding signals induce intracellular nuclear signaling and expression of effector molecules, and interact extensively in the pathophysiological response of NAFLD. It plays an essential role in promoting the innate immune response (17).

While innate immune cells are responsible for the initiation of liver damage, adaptive immune cells play a key role in the chronic inflammation as a result of ongoing liver damage (18). Liver inflammation is characterized by increased lymphocyte recruitment to the liver (19). In addition, innate immunity responds quickly to external substances such as antigens (20), whereas adaptive immunity specifically recognizes antigens processed and presented by antigen presenting cells and acquire antigen-specific memory response. Lymphocytes involved in the adaptive immune system include conventional T-cells (CD4, CD8, and γδ T cells), nonconventional regulatory T-cells (NKT and MAIT cells), and B-cells. Recently, studies on the relationship between NAFLD pathology and the adaptive immune system have been investigated. It has been reported that γδ T cells are recruited to the liver during NAFLD and upregulate the expression of interleukin (IL)-17 (21). Moreover, the increased infiltration of CD4+ T cells further promotes the induction of NAFLD, inflammation, and the progression of intrahepatic fibrosis. However, dysregulation of lipid metabolism during NAFLD may cause selective loss of CD4+ T cells and promote the development of HCC (22). CD8+ cytotoxic T cells are known to play a key role in the conversion of hepatic steatosis to NASH in the progression of NAFLD (23). NKT cells have been reported for immediate secretion of large amounts of cytokines such as interferon γ and IL-4 after presenting lipid antigens, affecting NAFLD in a manner similar to CD8+ T cells (24). In addition, the increased B-cell invasion is related to IFNγ, IgG levels, and NASH progression in the pathological association of NAFLD (25, 26). However, further research is still required to understand their precise role in NAFLD pathogenesis.

Both innate and adaptive immune cells are crucial for the maintenance of liver homeostasis. Liver damage can disrupt homeostasis of immune cells, resulting in marked changes in their composition and localization (27). In NAFLD, Kupffer cells (KCs) and monocyte-derived macrophages are key players in innate immunity (28). After steatosis development, KCs secrete chemotactic substances such as chemokine C-C motif ligands (CCL) 1, 2, and 5, which facilitate the infiltration of monocytes. Recruited monocytes then secrete large amounts of pro-inflammatory cytokines and promote hepatic steatosis to fibrosis progression (29). This aggravated inflammation leads to increase lymphocyte recruitment to the liver (30). These signals interact extensively with each other to form an integrated immune network in the pathophysiology of NAFLD. In this review article, we focus on describing the role and molecular pathways of KCs and macrophages in NAFLD and potential therapeutics targeting liver macrophages to treat NAFLD.

## 2 Macrophage subsets identified in the healthy and disease liver

Macrophages reside in various tissues and circulating blood and play a pivotal role as the first barrier to pathogenic invasion. In the liver, macrophages are divided into two major subsets. First are the monocyte-derived liver macrophages (MoMFs) derived from bone marrow hematopoietic stem cells. These MoMFs are recruited to the inflammatory site and are differentiated to produce the inflammatory mediators mainly under the influence of the microenvironment. The second subsets are KCs from yolk sac-derived erythromyeloid progenitor cells. KCs are locally proliferating, self-renewing, and phagocytic cells (31, 32). Macrophages are also divided into two phenotypes: classically activated macrophages (M1) and alternatively activated macrophages (M2). M1 is involved in Th1 responses, produces inflammatory cytokines (IL-1β, TNF-α, and IL-6) and toxic effector molecules (ROS and NO), and is characterized by the IL-12^hi^IL-23^hi^IL-10^lo^ phenotypes. M2 participates in polarized Th2 responses and is involved in tissue remodeling, dampening of the inflammation, allergies, and immunomodulation, and is characterized by the IL-12^lo^IL-23^lo^IL-10^hi^TGF-β^hi^ phenotype (33).

Recently, single cell sequencing analysis was performed to precisely identify different M1 and M2 macrophage populations. It helps to understand specific markers to identify specific macrophage subsets. Moreover, differences in scavenger receptors can distinguish the two populations. A common M1 macrophage marker is MRC1, whereas C-type lectin receptors and MARCO are common markers for the M2 macrophage population. Profile of differential gene expression in a single cell RNA sequencing analysis can determine characteristics of the different subsets. CD5L, VSIG4, SLC1A3, CD163, FOLR2, TIMD4, GFRA2, ADRB1, TMEM25, SLC40A1, HMOX1, SLC16A9, VCAM1, and SUCNR1 were proposed as key gene signatures specific to KCs (34). To distinguish the presence of recruited macrophages, two subsets were purified based on CLEC4F and TIM4, and bulk RNA-Seq was performed. As a result, Spp1, Chil3, Ccr2, and Gpr183 were identified as CLEC4F-macrophages. A group of genes specific to resident KCs was identified, including Timd4, Cd163, C6, Xlr, and Marco. It would be interesting to perform additional comparisons between monocytes-KC and resident-KCs (35).

KCs in response to lipid overload during NASH has been analyzed by niche-specific reprogramming studies of epigenetic landscapes and spatial proteogenomic studies (36–38). Moreover, MoMFs can acquire distinct phenotypes across different disease models (38). While monocytes entering the liver can differentiate into monocyte-derived KCs in NASH, non-KC macrophages are generated in other models. In hepatic inflammation, Mo-MFs can be differentiated into lipid-associated macrophages (LAM). Importantly, LAM have been found in the obese and cirrhotic human liver. Given the conserved gene signatures in recruited macrophages and a subset of KCs, it is possible that LAM may be a bona fide macrophage subset or rather an activation state of macrophages.

The hepatic immune response is driven by homeostatic regulation within the hepatic sinusoids, where KCs are located as mononuclear phagocytes. In the healthy liver, KCs are mainly located near the portal venous and arterial circulation and exert phagocytic functions (39). However, upon the hepatic inflammatory condition, macrophages mediate cellular crosstalk by facilitating communication between the liver and intestinal substances and function as the body’s first line of defense. Moreover, other immune cells such as neutrophils and NK cells form a network together and respond to inflammatory insults (40, 41).

## 3 Role of KCs and macrophages in NAFLD development

The liver responds to pathological changes in the body and induces hepatic inflammation promoting various cytokines and liver toxicity levels (42). Histological studies indicate that liver damage is accompanied by the abnormal appearance of hepatocytes and clusters of KCs in the liver (43). Fatty diet leads to lipid droplets in hepatocytes and a change in cellular composition present in the liver (44). KCs are involved in the production of reactive oxygen species (ROS), leading to hepatic oxidative stress and inflammation. KCs also activate hepatic stellate cells (HSC) involved in liver fibrosis (45). Activated KCs release various inflammatory cytokines, including ROS and growth factors. KCs exert these effects through direct cell-to-cell contact with hepatocytes. Recently, KCs have been shown to play a role in regulating the induction of CXCR2, a chemokine receptor in hepatocytes (46).

In different stages of liver diseases, resident KCs and MoMFs contribute to regulating hepatic inflammation, fibrogenesis, and HCC (47). As KC-specific markers, CLEC4F (C-Type Lectin Domain Family 4 Member F) and TIM4 (T-cell immunoglobulin and mucin domain containing 4) were used to identify KCs in the liver (48, 49). In addition to liver-resident KCs, infiltrated macrophages are distinguished by the expression of cell surface markers. High levels of F4/80 and low levels of CD11b were identified in liver-resident KCs, and high CD11b and low levels of F4/80 were identified in infiltrated macrophages (50, 51). In addition, CD68, a scavenger receptor for macrophage lipoprotein, was used as a representative macrophage marker (52). Ionized calcium binding adapter molecule 1 (IBA-1) was used to distinguish invasive macrophages (53).

Notably, an increased number of KCs/macrophages was observed in the liver where large lipid droplets were formed and positively correlated with the severity of NAFLD disease (43, 44). A large number of activated macrophages was also observed in the spaces between damaged hepatocytes in patients with NAFLD (54). During NAFLD to NASH progression, there was an increase in the accumulation of endolysosomal lipids in KCs, suggesting a key role of lipids for KC activation and their impact on NAFLD progression (55). Taken together, KCs play a role in fibrosis development upon lipid accumulation, and inflammation in the liver. KCs depletion resulted in suppressing HSC activation and fibrosis development in a fibrosis mouse model (56, 57).

The immune system altered by gut microbiota can exacerbate endotoxemia in NAFLD (58). KCs increase the endotoxin sensitivity in the liver through upregulation of CD14 caused by leptin changes (59, 60). Thereby endotoxin promotes the development of steatohepatitis by activating KCs through TLR4 and generating proinflammatory cytokines and reactive oxygen species (61). Mitochondrial DNA damage and the release of damage-associated molecular patterns (DAMPs) from dying hepatocytes activate TLR9 in KC-rich zones to trigger an inflammatory cascade (62, 63). This innate immune response of KCs leads to CD4+ T cells recruitment and increases T cell resistance (64). In addition, it is known that macrophages other than KCs also contribute to the pathogenesis of NAFLD.

## 4 Role of macrophages for insulin resistance in NAFLD

The relationship between abnormal changes in the intestinal microbiota and KCs has been extensively investigated as described above. Given that hepatic endotoxin is related to specific factors induced by leptin, leptin can affect insulin resistance. Increased leptin levels lead to overexpression of CD14 through activation of STAT3 signaling in KCs. It also causes an inflammatory response of the liver to bacterial endotoxin in the intestine and progresses from simple steatosis to steatohepatitis and liver fibrosis (60, 65). Leptin is one of the most prominent adipokines and binding of leptin to its receptor displayed on KCs can promote fatty acid oxidation and exacerbate liver inflammation and fibrosis in NAFLD (66). Leptin can also induce inflammatory responses by enhancing LPS-induced TNF production *via* the JNK/MAPK and p38 Pathway. KCs induced the up-regulation of TGFβ1-related connective tissue growth factors in a leptin receptor-dependent manner for activation of STAT3 and NF-κB. This increased the expression of genes related to fibrosis, such as collagen-I, tissue inhibitor of matrix metalloproteinases-1 (TIMP1), transforming growth factor β1 (TGFβ1), and connective tissue growth factor (CTGF/CCN2) along with HSC activation (67). Taken together, these results suggest that leptin-mediated signaling in KCs plays a role in inducing pro-fibrotic genes and activating HSC for fibrosis progression.

Adipose tissue macrophages (ATMs) increase the recruitment of blood-derived monocytes during NAFLD, and activation of apoptosis-related macrophages further promotes liver damage by recruiting neutrophils. This causes lipolysis-mediated lipotoxicity and the liver damage (68, 69). Both ATM and liver-resident macrophage KCs play an important role in the pathogenesis of obesity, diabetes, and NAFLD. ATM significantly secretes anti-inflammatory cytokines such as IL-10 that contribute to insulin resistance, impairs phosphorylation through insulin receptor substrate 1 (IRS1) and PI3K/AKT pathway related to IRS1, and reduces hepatocyte responsiveness to insulin (70, 71). In NAFLD, macrophage infiltration of adipose tissue is a key activity during the cross talk between adipose tissue and liver. Infiltrated macrophages to the adipose tissue are initially increased by proliferation of KCs in response to a high-fat diet. However, the subsequent increase of macrophages continues in response to the chemotactic signals induced by adipose tissue under the inflammation.

**Figure 2:**
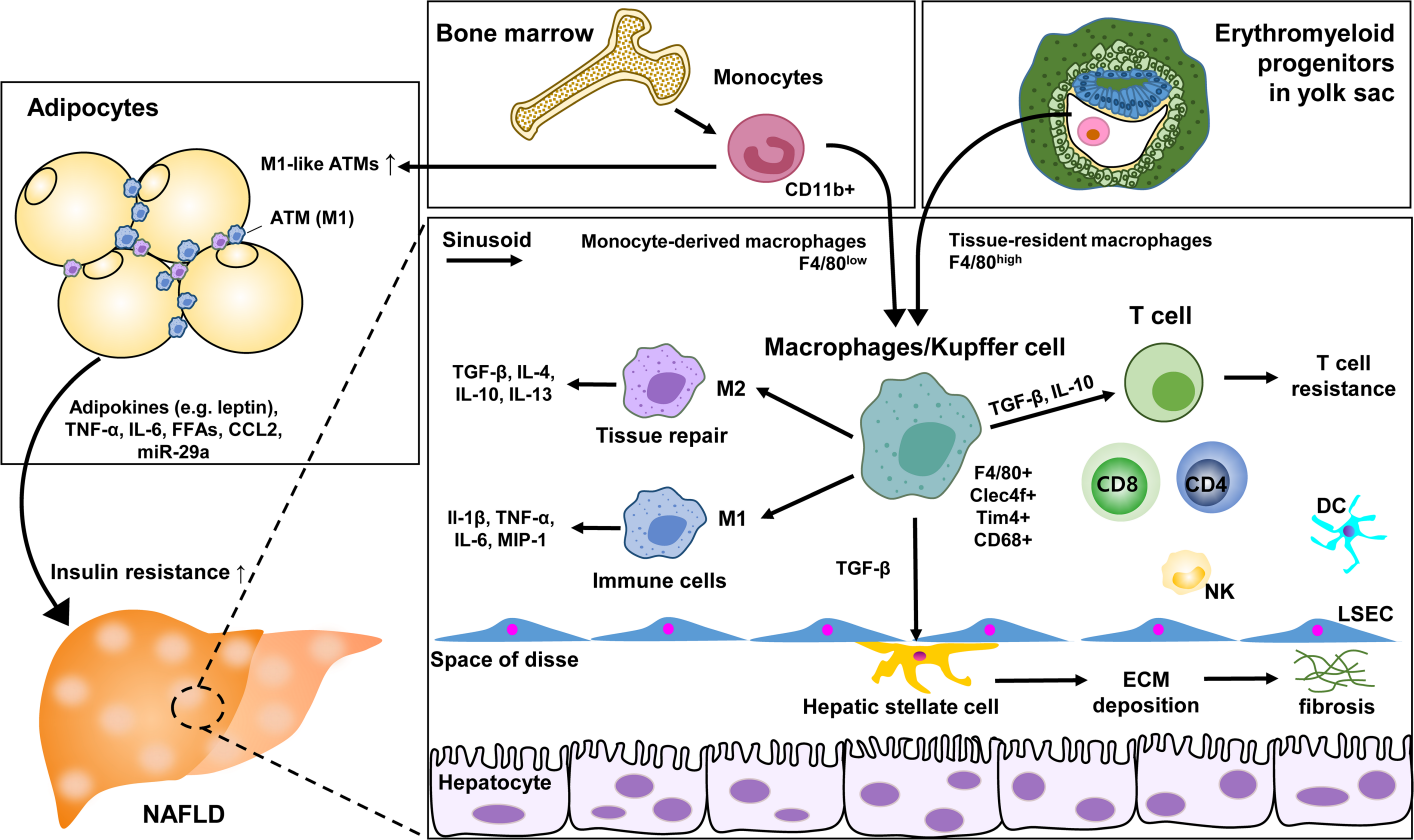
Role of KC and macrophages in NAFLD. ATM, adipose tissue macrophage; LSEC, liver sinusoidal endothelial cells; ECM, extracellular matrix; DC, dendritic cell; NK, natural killer cell.

Specific ATM subsets contributing to undergo phenotypic changes have been identified with immunostaining studies. CD206+CD11c+ appeared to be distributed among adipocytes rather than vascular lymphocyte clusters as markers (72). With molecular mechanisms involved in the impact of macrophages on insulin resistance, a high-fat diet increased the expression of epidermal growth factor receptor (EGFR) and its ligand amphiregulin in ATM. The inhibition of EGFR reduced the development of obesity and insulin resistance. This suggests that ATM EGFR activation plays an important role in adipose tissue and insulin dysfunction (73). Additionally, exosomal miR-29a transported by ATM has been reported to regulate obesity-related insulin resistance, with PPAR-γ as a downstream target (74). ATM’s cyclooxygenase-2 (COX-2) limits adipose tissue dysfunction in obese mice. As a result, the risk of monocyte infiltration, ATM proliferation, proinflammatory cytokines, and fibrosis was further magnified through the prostaglandin E2 (PGE2)/EP4 signaling (75). These ATM studies provide an association between metabolic disease and severity of NAFLD and strategies for potential therapeutic intervention (**Figure 2**).

## 5 Molecular mechanism for NAFLD by KC and macrophages

Several molecular signaling pathways in KCs and macrophages have been reported to contribute to NAFLD progression. These pathways include cell surface receptor to bind lipid moiety and modulate cellular response and intracellular signaling proteins to induce specific transcription factors for cell proliferation and inflammation. The understanding of molecular mechanisms is imperative to develop therapeutic targets designed to promoting/inhibiting these pathways. Moreover, macrophage polarization differences can lead to the repression or activation of various cytokines; thus the understanding of molecular pathways that promote the polarization of macrophages into a proinflammatory state or an anti-inflammatory state are of great interest in NAFLD (76).

As a cell surface receptor, the macrophage scavenger receptor (MSR1) has been shown to correlate with NAFLD such that mice deficient in MSR1 were protected from liver damage. MSR1 provides lipid intake of macrophages. MSR1 regulates JNK which in turn activates pro-inflammatory macrophages when lipopolysaccharide was not present. MSR1 deficient macrophages showed reduced TNF-a and IL-6. With these cytokines being crucial in inflammation, it makes sense as to how their dysregulation would affect NAFLD progression (44).

Recent studies revealed that M2 macrophages lead to the autophagy of HSC by releasing prostaglandin E2 (PGE2) and binding to the receptor EP4 on the exterior of HSCs. HSC activation is important in the development of liver fibrosis and cirrhosis. Autophagy of HSC results in the activation of HSC, converting them into myofibroblast and promoting NAFLD progression. Additionally, M2 macrophage polarization is involved in activating HSC, liver fibrosis, and extracellular matrix breakdown. It occurs through Akt/mTOR-independent and Erk 1/2 pathway where PGE2/EP4 signals lead to the autophagy of HSC (77).

As an intracellular signaling pathway, the cAMP-PKA-STAT3 signaling pathway is crucial for converting KCs to an M2 polarization. STAT3 plays important anti and pro inflammatory roles in the progression of NAFLD and acts as a protector from lipotoxicity. NAFLD mice with treatment of the drug Liraglutide showed reduced liver inflammation as well as reduced inflammatory features of KCs (43, 77). Retinol-binding protein 4 (RBP4) was abundantly found in patients diagnosed with NAFLD. RBP4 turned on the NF-kB signaling of KCs and elevated the amount of reactive oxygen species present. The activation of NF-kB pathway leads to an abundant amount of proinflammatory mediators as well as rallying of leukocytes. Coimmunoprecipitation assays and additional validation techniques revealed that NOX2 was activated by RBP4. NOX2 is significant in NAFLD due to its high expression levels in the liver. NOX2 is involved in generating high quantities of ROS and when highly activated, NOX2 affects immune functions and can lead to the progression of NAFLD. TNF-α enhanced the abundance of RBP4. KCs polarization contributes to the progression of NAFLD into hepatocellular carcinoma (HCC). RBP4 caused KCs to exist in an M1 polarization through activation of NOX2 and NF-kB pathways. Generation of triglycerides in hepatocytes was affected upon this polarization where TNF-α led to the initiation of the JAK2/STAT3 signaling pathway (78, 79).

Studies from NAFLD mice revealed the role of the long non-coding RNA SNHG20 in the progression of NAFLD. Silencing of long non-coding RNA prevented NAFLD by hindering the polarization of KCs. Macrophages were analyzed and upon silencing this RNA, the M1 polarization was restricted. Similarly, overexpression of this RNA resulted in M2 polarization. STAT6, essential in turning on M2 macrophages, was shown to be activated by the overexpression of SNHG20. Results revealed that the progression of NAFLD to HCC relied on this STAT6 activation (80).

**Figure 3:**
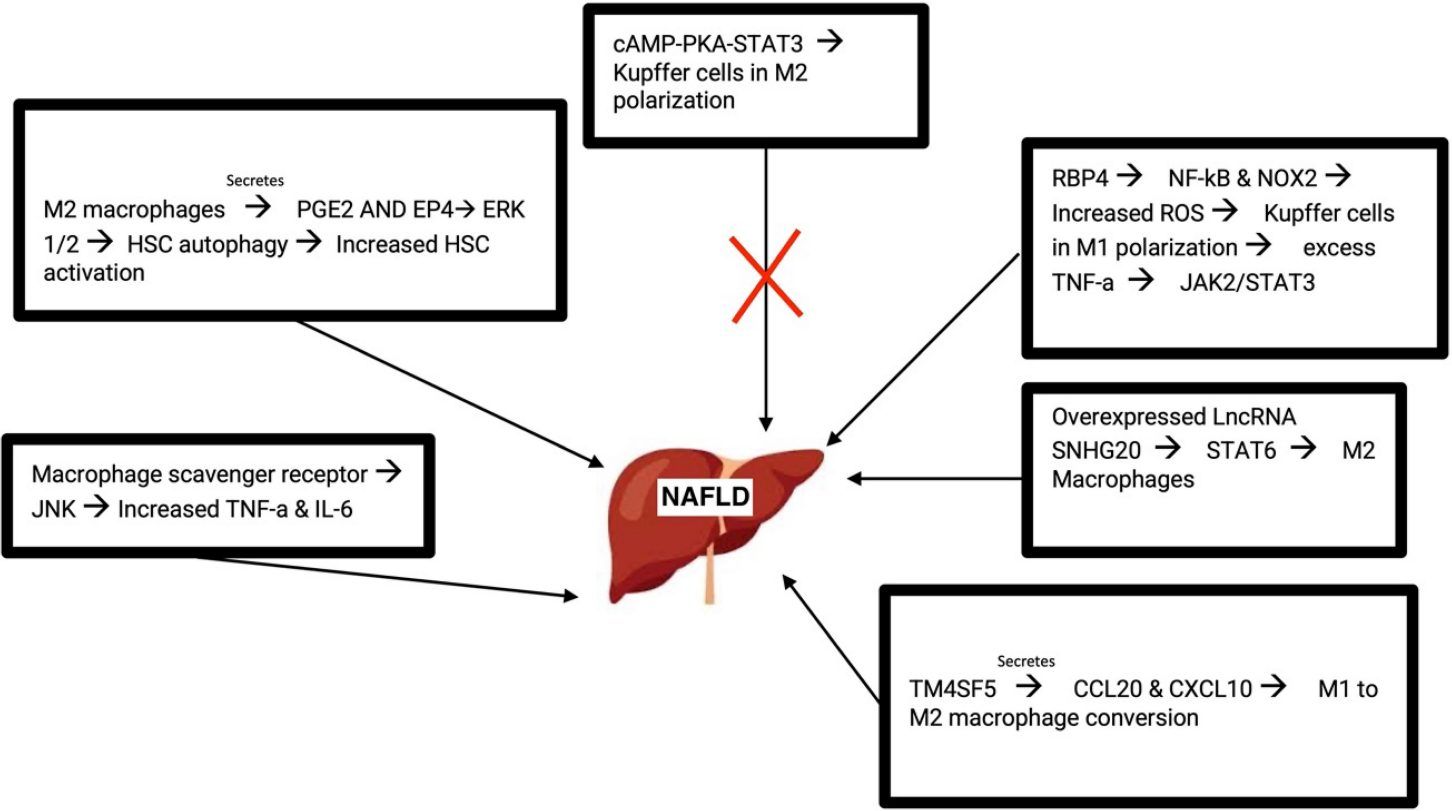
Molecular pathways that lead to the development of NAFLD. M, macrophage; PGE2, prostaglandin E2; HSC, hepatic stellate cell; RBP4, retinol-binding protein 4; ROS, reactive oxygen species; LncRNA, Long non-coding RNA.

TM4SF5 is a member of the tetraspanin family. TM4SF5 communication between hepatocytes and macrophages leads to the production of the chemokines CCL20 and CXCL10. TM4SF5 macrophages lead to M1 activation and assist with glucose uptake and glycolysis. Thus, it is likely that TM4SF5 expression in macrophages affects the inflammatory environment midst the progression of NAFLD (81) (**Figure 3**).

## 6 Therapeutics targeting KCs and macrophages in NAFLD

Macrophage polarization plays a key role in NAFLD progression due to the secretion of pro-inflammatory cytokines. Therefore, targeting major pathways involved in the immune response that lead to these polarization differences are promising therapeutic treatments (82). Potential NAFLD therapeutics currently being developed are described below.

Rhubarb free anthraquoines (RFAs) were found to function in inhibiting NLRP3 inflammasome signaling, and ultimately ameliorate NAFLD in mice. RFAs were found to inhibit the inflammasome by preventing both its transcription and its assembly. By treating KCs with RFAs, the authors were able to see reduced expression of IL-1B, and liver histology’s of mice suffering from NAFLD treated with RFAs had less fibrosis than the controls. By targeting the inflammasome in KCs, researchers are able to ameliorate the effects of NAFLD (83).

Macrophage polarization is one of the key aspects involved in the progression of NAFLD. With increased pro-inflammatory cytokines being linked to increased lipogenesis, understanding what drives macrophages to polarize into a more pro-inflammatory state is a viable method to target the progression of NAFLD. The enzyme 17B-HSD7 is expressed in high amounts in M1 macrophages (pro-inflammatory type macrophage). Deletion of 17B-HSD7 in knockout mice who were fed a high fat diet to induce NAFLD led to less accumulation of lipids and showed improvement in NAFLD mice. Deletion of 17B-HSD7 led to less M1 macrophage polarizations by inhibiting NLRP3 inflammasome. Treating with fenretinide to inhibit 17B-HSD7 led to the same findings as performing the knockout of 17B-HSD7 resulting in less pro-inflammatory cytokines present due to a decrease in the M1 macrophages population (84).

XBP-1, a transcription factor observed in macrophages, helps enhance inflammatory cytokine production. XBP-1 has also been shown to activate NLRP3 in steatohepatitis. The expression of XBP1 was measured in human liver samples and its deficiency was found to improve NAFLD progression. Macrophage activation was found to be regulated by XBP1, where XBP1 directly inhibited BNIP3 preventing it from activating the STING pathway and ultimately NLRP3 activation. The deficiency in XBP1 led to more mitophagy activation in macrophages, preventing the macrophages from becoming activated due to the presence of pro-inflammatory cytokines like IL-1B and TNF-a. Mice treated with toyocamycin, an XBP1 inhibitor, showed improved liver fibrosis. The authors of this study suggested that inhibiting XBP1 is an effective way to inhibit the STING pathway which has been shown to cause NAFLD (85).

Losartan is an antagonist for the angiotension II receptor and was previously found to stop NASH disease severity. Wang et al. demonstrated that treating NAFLD mice with losartan not only lowered lipogenesis and enhanced mitochondrial biogenesis, but it also resulted in a decrease amount of M1 type macrophages present. The mechanism by which losartan decreased M1 polarization was by inhibiting HIF-1a, a transcription factor that has been found to be upregulated in patients with NAFLD. By inhibiting HIF-1a with losartan, less lipid accumulation occurs resulting in less triggers for macrophage polarization into the M1 type which is known to be pro-inflammatory (82).

Epigallocatechin-3-Gallate (EGCG) is commonly found in green tea and has been linked to being involved in anti-inflammatory and immunomodulatory responses. In a recent study from 2021, the authors treated NAFLD mice who were fed a high fat diet with EGCG. Treated mice were found to have lower fat accumulation in the liver and even prevented liver damage in mice with NAFLD. EGCG treated mice were also analyzed *via* flow cytometry for differences in M1/M2 macrophage polarization, and results showed that mice with EGCG had decreased M1 macrophages and increased M2 macrophages. Authors suggested that the abundancy of these M2 macrophages may be involved in secreting various cytokines to ameliorate NAFLD mice (86).

CCR2-CCR5 dual antagonist has recently been reported in several studies as a substance that can improve NASH and NAFLD (87). In patients with NAFLD, CCL2 levels of serum and hepatic mRNA were elevated, which increased the recruitment of CCR-positive bone marrow-derived monocytes into the liver. This affected the development of hepatic inflammation, fibrosis, and steatosis. Thus, genetic deletion of CCR2 not only improved NAFLD but also improved insulin resistance. In addition, the production of CCL5 in liver macrophages is related to lipid accumulation, and it binds to CCR5 in HSC to progress liver fibrosis. Based on this finding, cenicriviroc (CVC), a CCR2-CCR5 dual antagonist, was expected to improve NASH and NAFLD and was tested in clinical trials (88).

Galectin-3 (Gal-3) is a β-galactoside binding protein and is secreted by macrophages. Gal-3 is activated in response to tissue damage and is associated with several disease pathogenesis including chronic inflammation and fibrogenesis (89). Belapectin, an inhibitor of Gal-3, has been shown to improve and prevent fibrosis progression in animal studies of toxin-induced liver fibrosis. Contrary to the animal studies, in NASH patients without pre-existing cirrhosis, Gal-3 inhibitor showed an improvement of hepatic venous pressure gradient with no effect on fibrosis (90). The protective effect of this drug is currently underway in patients with NASH cirrhosis without preexisting severe diseases (**Table 1**).

**Table 1:** Therapeutics targeting kupffer cells or macrophages in NAFLD.

Therapeutic	Target	References
Rhubarb free anthraquoines (RFAs)	Inhibits NLRP3 inflammasome signaling in Kupffer cells	(83) Wu, C et al. *J. Transl. Med 2022, 20 (1), 294*
Fenretinide	Inhibits the enzyme 17B-HSD7 thus inhibiting NLRP3 inflammasome signaling and inhibiting M1 macrophages	(84) Dong, X et al. *Acta Pharm. Sin. B 2023, 13 (1), 142–156*
Toyocamycin	Inhibits XBP1, which in turn inhibits the STING pathway and NLRP3 activation	(85) Wang, Q et al. *JHEP Rep. 2022, 4 (11), 100555*
Losartan	Inhibits HIF-1a which leads to less M1 macrophage polarizatiion	(82) Wang, C.-H et al. *Int. J. Mol. Sci. 2021, 22 (15), 7841*
Epigallocatechin-3-Gallate (EGCG)	Leads to the production of more M2 macrophages and less M1 macroophages	(86) Du, Y et al. *Nutrients 2021, 13 (2), 599*
CCR2/CCR5 antagonist	Inhibits CCL2 and CCL5 in liver macrophages	(87) Nagata, N et al. *Medicina. 2022, 58(6),761* *(* 88) Ratziu, V et al. *Int. J. Mol. Sci. 2022, 23(12), 6696*
Galectin-3 (Gal-3) inhibitor	Inhibits Gal-3 which is secreted by macrophages	(89) Wiering, L et al. *J Endocrinol. 2023, 256(1)*, e220194 (90) Chalasani, N et al. *Gastroenterology 2020, 158(5), 1334-1345*

## 7 Conclusion

The understanding of NAFLD pathogenesis has been significantly advanced with new insights of KCs and macrophages into NAFLD progression. KCs, the resident macrophages of the liver, are migrated and activated by hepatocyte damage, and the activation of KCs are reversible depending on the hepatic inflammatory status. KCs appear to be involved at each stage of NAFLD progression. Moreover, KCs contribute to fibrosis and carcinogenesis by promoting inflammatory responses that cause hepatocellular damage and activate HSCs. Although they play a key role in the induction phase of NAFLD, additional systematic studies are necessary for elucidating KCs activation signals and their function on NAFLD pathogenesis. Future studies identifying factors to control the polarization of M1 to M2 KCs would help design therapeutics to interfere with M2 polarization. In addition, the role of KCs in the development of NASH and liver disease through inflammatory cell death process and pyroptosis has recently been discussed (91). These studies provide additional information on the etiology of NAFLD and the development of therapeutics. Recently, various natural products and drugs have been considered for potential therapeutic agents targeting KCs or macrophages to treat NAFLD, including RFAs, Fenretinide, Toyocamycin, Losartan, EGCG, CCR2/CCR5 antagonist, and Gal-3 inhibitor. Since most of these are limited to *in vivo* animal studies, additional clinical studies are required for treatment of NAFLD patients. Ultimately, lifestyle changes such as weight control by proper diet and exercise are the most reasonable recommendations for reducing NAFLD.

## Author contributions

S-JP contributed to manuscript research and writing. JD contributed to manuscript research and writing. EU contributed to manuscript writing and review. YH contributed to manuscript supervision, writing, and review. All authors contributed to the article and approved the submitted version.
